# Parental smoking is associated with adolescent loneliness: evidence from 71 low- and middle-income countries

**DOI:** 10.3389/fpubh.2025.1497136

**Published:** 2025-02-26

**Authors:** Jintang Xie, Hui Yang, Min Zhao, Costan G. Magnussen, Bo Xi

**Affiliations:** ^1^Department of Epidemiology, School of Public Health, Qilu Hospital, Cheeloo College of Medicine, Shandong University, Jinan, China; ^2^Department of Nutrition and Food Hygiene, School of Public Health, Cheeloo College of Medicine, Shandong University, Jinan, China; ^3^Baker Heart and Diabetes Institute, Melbourne, VIC, Australia; ^4^Research Centre of Applied and Preventive Cardiovascular Medicine, University of Turku, Turku, Finland; ^5^Centre for Population Health Research, University of Turku and Turku University Hospital, Turku, Finland

**Keywords:** loneliness, adolescent, tobacco use, mental health, parental smoking

## Abstract

**Introduction:**

The association between parental smoking and adolescent mental health is not well understood. We aimed to examine the association between parental smoking and adolescent loneliness using global data collected from the Global School-based Student Health Survey (GSHS) between 2009 and 2019.

**Methods:**

A total of 187,552 adolescents aged 12–15 years in 71 low- and middle-income countries (LMIC) from the GSHS with cross-sectional design were included in this study. Loneliness was defined as feeling lonely sometimes or more frequently in the past 12 months. Parental smoking, reported by the adolescent, was defined as the use of any form of tobacco by the father and/or mother. Logistic regression models were used to examine the odds ratio (OR) of adolescent loneliness according to parental smoking status.

**Results:**

Compared with neither parent smoking, parental smoking was associated with adolescent loneliness (father only: OR = 1.18, 95% CI = 1.10–1.26; mother only: OR = 1.39, 95% CI = 1.15–1.67; both: OR = 1.65, 95% CI = 1.41–1.93) after adjustment for potential covariates. Subgroup analyses stratified by sex, age, and WHO region showed similar results (except not in the African and Western Pacific regions).

**Discussion:**

Parental smoking is associated with loneliness in adolescents from LMIC. Our finding expands the well-known physical damage of parental smoking in adolescents to the psychological damage. Strict policies and strategies should be established to encourage smoking cessation for parents who are current smokers.

## 1 Introduction

Secondhand smoke exposure among adolescents has been identified as a major modifiable risk factor for morbidity and mortality (1). Globally, the proportion of adolescents exposed to secondhand smoke at any place on more than 1 day in the past 7 days exceeded 60% based on data from 142 countries/territories in 2010–2018, with more than 30% of adolescents exposed to secondhand smoke at home (2). Parental smoking is the main source of secondhand smoke exposure at home (3). It has been shown that adolescents with parents that smoked were more likely to start smoking (4, 5). In addition, parental smoking has been associated with adverse effects on adolescent health such as an increased likelihood of elevated blood pressure, obesity, cardiometabolic risk in childhood, respiratory symptoms, reduced lung function, and multiple sclerosis during adolescence (6–11).

Parental smoking not only increases physical health risk but also contributes to psychological harm. A study involving six European countries found that maternal smoking was associated with inattention and hyperactivity of their offspring, and another study of 617 families found that parental smoking was associated with an increased risk of disruptive behavior disorders, preference for risk taking, and aggressive attitudes (12, 13). Loneliness is defined as a painful encounter when the need for a person's intimacy is not met, or when a person's social network does not match his/her preference in terms of number or attributes. A study based on data from 25 countries showed that more than 18% of middle-school students reported feeling lonely most of the time or constantly (14). Another survey based on data from 37 countries indicated that the prevalence of loneliness among adolescents aged 15–16 years had nearly doubled in 2018 (30.9%) since 2012 (17.1%) (15). Adolescent loneliness has been identified as a significant risk factor for mental health disorders and suicidal ideation during adolescence and mental illness in adulthood (16–19). Epidemiological studies have assessed exposure to environmental tobacco smoke as a risk factor for a variety of behavioral and neurodevelopmental disorders in children (20, 21). In addition, maternal smoking during pregnancy is associated with mental deficits and behavioral problems in childhood (13). McCarthy et al. (22) also found that nicotine exposure has negative effects on the developing brain and has lasting effects on brain structure and neurotransmitter signaling. Although secondhand smoke exposure has been shown to be associated with adolescent loneliness (23), the association between parental smoking, as a proxy of secondhand smoke exposure, and adolescent loneliness remains unclear.

Using data from the Global School-based Student Health Survey (GSHS) conducted in 2009–2019, we aimed to assess the relationship between parental smoking and feelings of loneliness in adolescents aged 12–15 years from 71 low- and middle-income countries (LMIC).

## 2 Methods

### 2.1 Data source

**Figure 1:**
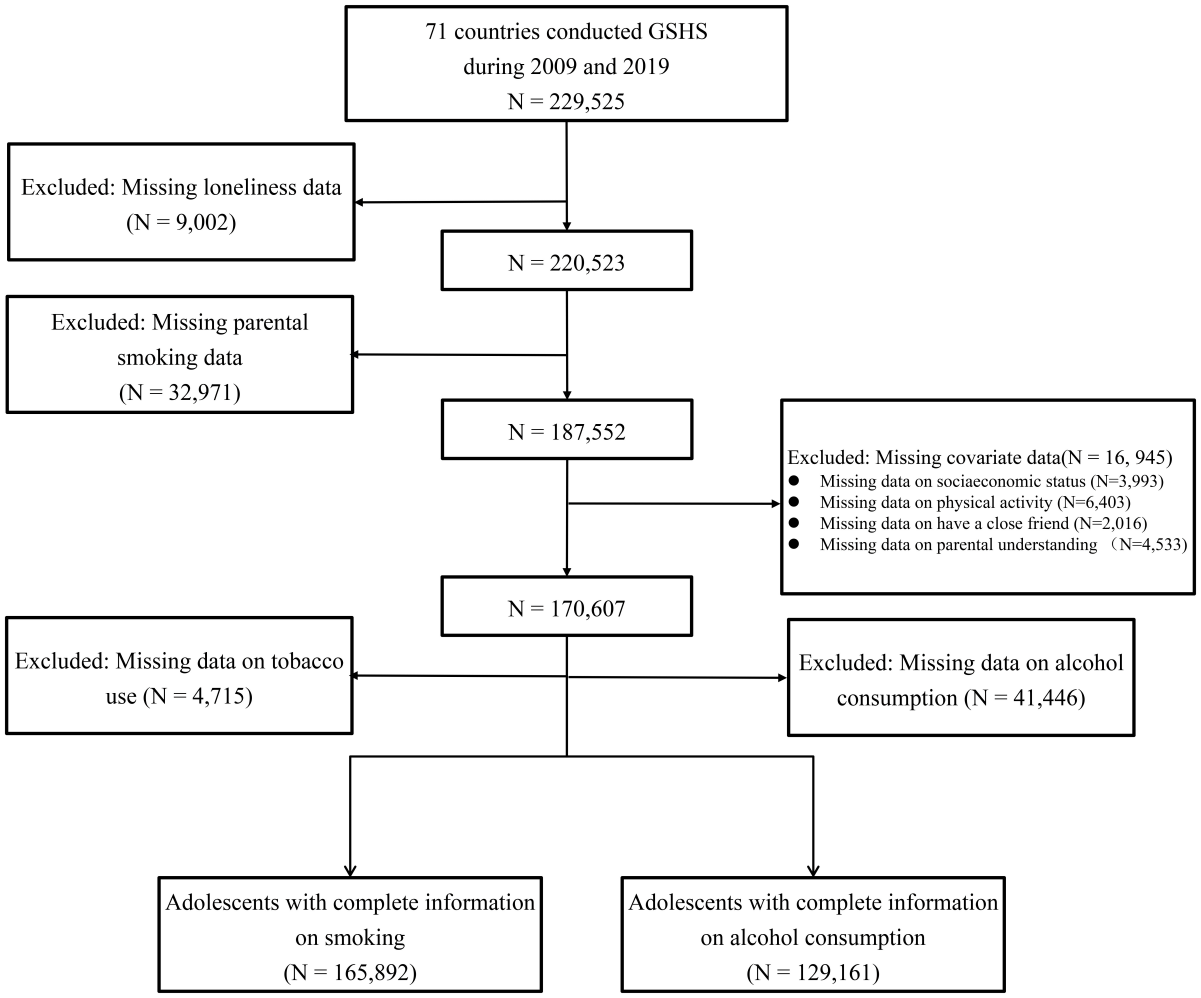
Flowchart of inclusion and exclusion of study participants. GSHS, Global School-based Student Health Survey.

The data were obtained from the GSHS, which was jointly developed by the WHO, the US Center for Disease Control and Prevention (CDC), and other United Nations allies. This survey is conducted in LMIC and follows standardized procedures to randomly select a study sample from schools. The aims of GSHS are to collect data on adolescents' health behaviors using the same standardized two-stage sampling methodology in each participating country. In the first stage, schools were randomly selected with a probability proportional to their enrolment size. In the second stage, classes with students in the targeted age groups (ages 12–15 years old) were also randomly selected. All students from each selected class were invited to fill in the structured questionnaire during normal school hours. The country-specific self-administration questionnaire consists of core questionnaire modules and country-specific questions. The questionnaires in English were translated into local language of each country and were pilot-tested for comprehension to ensure clarity for the participants. All responses were collected using self-administered, anonymous, computer-scannable forms to maintain privacy and facilitate efficient data collection. Participation was voluntary for all students and informed verbal or written consent was obtained from all students and their parents/guardians. A flowchart of exclusion/inclusion of the study participants is shown as Figure 1. A total of 187,552 students with complete information on parental smoking and loneliness were included in the study.

### 2.2 Assessment of adolescent loneliness

Following previous studies (14, 24), the measure of adolescent loneliness was based on responses to the following question: “How often have you felt lonely in the past 12 months?” with five independent options representing the experience of participants (never, rarely, sometimes, most of the time, always) available for selection. For data analysis, participants were dichotomized into two groups based on the responses: (1) no loneliness (never or rarely); and (2) affirmative loneliness (sometimes, most of the time or always). In sensitivity analysis, participants were re-classified into two groups: (1) no loneliness (never, rarely or sometimes); and (2) affirmative loneliness (most of the time or always).

### 2.3 Assessment of parental smoking

Parental smoking status was assessed by adolescent response to the question “Which of your parents or guardians use any form of tobacco?”, and the corresponding answers included “neither”, “my father or male guardian”, “my mother or female guardian”, and “both”.

### 2.4 Assessment of covariates

Covariates of sex, age, socioeconomic status, sufficient physical activity, having close friends, parental understanding, survey year, and country were adjusted in our regression models because they may confound the association between parental smoking and adolescent loneliness. Based on previous studies, low socioeconomic status measured by hunger due to a lack of food, was defined as getting hungry most of the time or always due to a lack of food (25, 26). Sufficient physical activity was defined as participants engaging in active exercises on at least 60 min per day (27). Having close friends was defined as having at least one close friend (14). Parental understanding was defined as parents or guardians understanding adolescents' problems or worries most of the time or always (24). Based on participants' responses to the questions “During the past 30 days, on how many days did you smoke cigarettes?” and “During the past 30 days, on how many days did you use any tobacco products other than cigarettes, such as pipe, hand-rolled tobacco, or chewing tobacco?”, we categorized tobacco use into never, 1–9 days, and ≥10 days. Similarly, based on participants' responses to the question “During the past 30 days, on how many days did you have at least one drink containing alcohol?”, we categorized alcohol consumption into never, 1–9 days, and ≥10 days (28). Hunger due to lack of food was not adjusted for in Bhutan as this variable was not collected. Information on physical activity was not collected in Maldives and Malawi. In Cambodia parental understanding was not adjusted as information on this variable was not collected. Myanmar did not collect information on tobacco use, so this variable was not included for adjustment in this country. Similarly, Afghanistan, Bahrain, Egypt, Iraq, Kuwait, Morocco, Pakistan, Palestine, Qatar, United Arab Emirates, and Yemen did not collect information on alcohol consumption, and therefore this variable was not considered for adjustment in these countries.

### 2.5 Statistical analysis

Strata, primary sampling units, and sampling weights were considered in all data analyses based on the methodology of GSHS. Proportion estimates and standard errors of basic characteristics in each country were calculated. A Chi-square test was used to compare the prevalence of adolescent loneliness across different parental smoking categories, overall and by subgroup (sex, age group, and WHO region group). After adjusting for potential covariates, multivariable logistic regression models were used to assess the relationship between parental smoking and adolescent loneliness using odds ratios (ORs) and their 95% confidence intervals (CIs). Model 1 was adjusted for age, sex, and hunger due to lack of food. Model 2 was additionally adjusted for having a close friend, parent understanding your problem, doing enough physical activity, survey year, and country. Furthermore, we conducted a subgroup analysis by the categories of tobacco use and alcohol consumption. We also conducted a mediation analysis using bootstrap method (with 1,000 simulations) to examine the mediation effect of adolescent tobacco use or alcohol consumption in association between parental smoking and adolescent loneliness. A sensitivity analysis was performed using a different definition of loneliness based on participants' responses (detailed earlier). A two-sided *P* < 0.05 was considered statistically significant. All statistical analyses were conducted using SAS 9.4 (SAS Institute, Cary, North Carolina, USA), while the mediation analysis was performed using R version 4.3.1 with the mediation package (version 4.5.0).

## 3 Results

### 3.1 Characteristics of study participants

Table 1 shows participant characteristics from 71 LMICs in five WHO regions surveyed from 2009 to 2019. A total of 187,552 adolescents aged 12–15 years with complete data on loneliness and parental smoking were included. Of these adolescents, 36.8% had at least one parental smoker, ranging from 7.1% in Panama to 76.6% in Tokelau; 39.6% reported feeling lonely sometimes or more frequently in the past 12 months, ranging from 17.9% in Tanzania to 67.8% in the Solomon Islands. Among those adolescents, 6.4% reported low socioeconomic status, 15.6% reported sufficient physical activity, 94.3% reported having at least one close friend, and 38.3% reported that their parent understood their problems. For tobacco use in the past 30 days, 90.0% reported no use, 7.8% used on 0–9 days, and 2.1% on 10 or more days. For alcohol consumption in the past 30 days, 90.0% reported no use, 9.2% drinked on 0–9 days, and 0.8% on 10 or more days (Supplementary Table S1).

**Table 1:** Characteristics of the Global School-based Student Health Surveys by country, 2009–2019.

**Country**	**Survey year**	**Sample size**	**Boys, %**	**Age range, years**	**Parental smoking^†^, %(SE)**	**Adolescent loneliness^‡^, %(SE)**	**Response Rate, %**
**Africa**							
Benin	2016	667	44.1	12–15	8.1 (2.5)	31.3 (3.5)	94.1
Ghana	2012	1,026	49.1	12–15	17.9 (2.9)	50.0 (2.7)	77.1
Liberia	2017	321	43.3	12–15	13.3 (2.7)	58.8 (3.3)	61.1
Malawi	2011	2,011	47.2	12–15	15.4 (2.1)	43.6 (2.0)	92.1
Mauritania	2010	1,093	45.0	12–15	21.8 (2.6)	33.7 (1.4)	86.1
Mauritius	2017	1,623	47.7	12–15	27.9 (1.8)	31.5 (2.1)	83.1
Mozambique	2015	596	49.5	12–15	11.0 (2.3)	29.9 (3.3)	92.1
Namibia	2013	1,247	41.5	12–15	24.7 (2.4)	57.2 (2.0)	65.1
Seychelles	2015	1,674	46.6	12–15	28.5 (1.5)	39.5 (1.4)	81.1
Tanzania	2014	2,425	44.8	12–15	10.5 (1.1)	17.9 (1.5)	94.1
**America**							
Anguilla	2016	502	47.8	12–15	12.3 (1.6)	35.8 (1.8)	90.1
Antigua and Barbuda	2009	1,037	45.8	12–15	15.9 (1.6)	41.9 (2.4)	87.1
Argentina	2018	34,100	47.6	12–15	34.7 (0.8)	39.9 (0.7)	94.1
Bahamas	2013	1,177	45.2	12–15	18.2 (1.5)	33.0 (1.5)	90.1
Barbados	2011	1,356	46.5	12–15	19.1 (1.1)	36.7 (1.7)	90.1
Bolivia	2012	2,563	49.4	12–15	15.0 (0.8)	32.5 (1.1)	93.1
British Virgin Islands	2009	1,101	44.0	12–15	9.6 (0.9)	33.5 (1.4)	92.1
Costa Rica	2009	2,170	47.8	12–15	15.3 (1.0)	26.7 (1.4)	96.1
Curacao	2015	1,399	46.4	12–15	22.4 (1.1)	39.5 (1.7)	94.1
Dominican Republic	2016	859	42.8	12–15	12.4 (2.0)	39.7 (2.6)	91.1
Guyana	2010	1,623	44.2	12–15	25.8 (1.8)	55.8 (2.3)	83.1
Honduras	2012	1,376	47.5	12–15	11.7 (1.3)	30.4 (1.6)	93.1
Jamaica	2017	894	43.7	12–15	25.5 (2.0)	46.6 (2.2)	85.1
Panama	2018	1,495	45.4	12–15	7.1 (0.8)	40.1 (1.6)	96.1
Peru	2010	2,084	47.9	12–15	13.8 (1.2)	38.4 (2.1)	89.1
Saint Lucia	2018	1,174	43.0	12–15	15.2 (1.2)	42.3 (2.0)	88.1
Saint Vincent and the Grenadines	2018	910	45.6	12–15	15.0 (1.5)	53.3 (1.9)	89.1
Suriname	2016	1,383	44.8	12–15	35.2 (1.8)	42.5 (1.9)	96.1
Trinidad and Tobago	2017	2,437	45.6	12–15	27.8 (1.8)	36.6 (1.7)	89.1
Uruguay	2012	2,773	47.1	12–15	37.8 (1.3)	25.7 (1.0)	97.1
**Eastern Mediterranean**							
Afghanistan	2014	1,351	36.8	12–15	17.7 (2.9)	48.3 (3.5)	93.1
Bahrain	2016	4,893	51.0	12–15	18.0 (0.8)	36.5 (1.5)	91.1
Egypt	2011	2,236	46.0	12–15	48.6 (2.3)	33.6 (2.5)	95.1
Iraq	2012	1,442	55.4	12–15	25.1 (1.2)	28.6 (2.1)	94.1
Kuwait	2015	1,777	46.0	12–15	33.9 (2.3)	40.3 (2.6)	88.1
Lebanon	2017	3,107	40.3	12–15	51.1 (1.5)	26.1 (1.2)	93.1
Morocco	2016	3,555	49.9	12–15	18.2 (1.2)	37.5 (1.0)	91.1
Pakistan	2009	4,676	74.5	12–15	28.2 (2.4)	38.1 (1.8)	94.1
Palestine	2010	12,994	45.4	12–15	41.0 (0.6)	31.0 (0.7)	95.1
Qatar	2011	1,410	42.0	12–15	23.1 (1.7)	37.9 (2.1)	80.1
Syria	2010	2,821	39.8	12–15	46.8 (1.6)	31.5 (1.8)	96.1
United Arab Emirates	2016	3,136	46.4	12–15	20.3 (1.3)	33.9 (1.3)	91.1
Yemen	2014	1,350	51.4	12–15	31.8 (2.8)	31.4 (2.2)	89.1
**South-East Asia**							
Bangladesh	2014	2,625	37.0	12–15	30.1 (2.5)	42.4 (2.3)	96.1
Bhutan	2016	2,558	40.3	12–15	42.1 (1.5)	59.1 (1.2)	78.1
Indonesia	2015	8,019	45.7	12–15	57.2 (0.8)	43.9 (1.0)	91.1
Maldives	2014	1,416	39.8	12–15	40.6 (1.8)	47.0 (1.7)	81.1
Myanmar	2016	2,117	45.7	12–15	44.6 (1.9)	41.7 (1.7)	95.1
Nepal	2015	4,294	45.3	12–15	33.7 (1.9)	31.0 (1.0)	94.1
Sri Lanka	2016	2,094	42.8	12–15	20.2 (1.8)	27.0 (2.1)	94.1
Thailand	2015	3,809	46.4	12–15	34.0 (2.2)	34.8 (1.7)	93.1
Timor-Leste	2015	1,079	43.1	12–15	39.0 (2.5)	38.4 (2.1)	69.1
**Western Pacific**							
Brunei Darussalam	2014	1,678	46.2	12–15	32.8 (1.4)	44.6 (1.4)	92.1
Cambodia	2013	1,755	44.4	12–15	35.7 (1.9)	46.7 (1.8)	97.1
Cook Islands	2015	314	47.1	12–15	47.4 (2.0)	35.2 (3.4)	87.1
Fiji	2016	1,243	48.0	12–15	36.9 (2.5)	45.9 (2.5)	82.1
French Polynesia	2015	1,741	49.4	12–15	42.9 (1.8)	35.2 (1.4)	92.1
Kiribati	2011	1,030	42.9	12–15	55.7 (1.9)	30.4 (1.4)	77.1
Laos	2015	1,610	42.1	12–15	45.4 (1.9)	27.4 (1.7)	98.1
Malaysia	2012	15,110	50.6	12–15	42.3 (1.0)	34.1 (0.7)	93.1
Mongolia	2013	3,616	47.6	12–15	45.7 (1.1)	37.9 (1.2)	98.1
Nauru	2011	230	41.3	12–15	60.7 (3.3)	56.7 (3.4)	64.1
Niue		69	49.3	12–15	28.6 (4.8)	42.0 (4.0)	72.1
Philippines	2015	5,765	43.7	12–15	40.2 (1.5)	59.9 (1.2)	94.1
Samoa	2017	923	33.3	12–15	29.2 (1.5)	26.5 (1.7)	88.1
Solomon Islands	2011	639	50.1	12–15	61.1 (5.3)	67.8 (3.5)	71.1
Tokelau	2014	49	59.2	12–15	76.6 (6.4)	52.6 (7.5)	60.1
Tonga	2017	1,926	46.7	12–15	40.8 (1.7)	34.2 (1.3)	94.1
Tuvalu	2013	575	47.7	12–15	44.1 (2.1)	19.9 (1.7)	85.1
Vanuatu	2016	773	38.7	12–15	38.5 (3.0)	53.0 (2.2)	61.1
Wallis and Futuna	2015	651	48.9	12–15	56.4 (2.6)	39.5 (1.4)	91.1
**Total**		187,552	47.0	12–15	36.8 (0.9)	39.6 (0.6)	81.7

*SE, standard error*.

^†^Parental smoking is defined as use of any form of tobacco products by the father or mother.

^‡^Sense of loneliness is defined as adolescent feeling lonely sometimes, most of the time or always in the past 12 months.

### 3.2 Proportions of adolescent loneliness across different categories of parental smoking status

The prevalence of loneliness among adolescents whose parents used any form of tobacco (father only: 41.0%; mother only: 45.6%; both: 49.4%) was higher than that among adolescents whose parents did not smoke (35.5%). The results were similar in the subgroup analyses by sex, age, and WHO region (Table 2). In the sensitivity analysis, we also observed similar results overall or in the subgroups according to the alternate definition of loneliness (Supplementary Table S2).

**Table 2:** Prevalence of adolescent loneliness across different categories of parental smoking stratified by sex, age, and WHO region.

	***N***	**Parental smoking**				***P*-value**
		**Neither**	**Father only**	**Mother only**	**Both**	
**Total, % (SE)**	187,552	35.5 (0.6)	41.0 (0.9)	45.6 (2.1)	49.4 (1.7)	< 0.001
**Sex**						
Boys, % (SE)	88,055	31.8 (0.7)	36.1 (1.2)	41.3 (2.8)	49.0 (2.7)	< 0.001
Girls, % (SE)	99,497	39.4 (0.8)	46.5 (1.3)	50.4 (2.9)	49.8 (2.2)	< 0.001
**Age groups, years**						
12–13, % (SE)	64,948	31.6 (0.9)	37.1 (1.3)	40.5 (2.9)	44.9 (2.7)	< 0.001
14–15, % (SE)	122,604	37.8 (0.6)	43.5 (1.1)	48.3 (2.8)	51.9 (2.3)	< 0.001
**WHO region**						
Africa, % (SE)	12,683	33.3 (1.4)	35.7 (2.6)	46.0 (7.1)	42.4 (5.0)	0.064
America, % (SE)	62,413	34.5 (0.9)	46.7 (1.7)	53.7 (3.1)	50.7 (3.2)	< 0.001
Eastern Mediterranean, % (SE)	44,748	31.7 (1.0)	35.4 (1.8)	40.1 (3.3)	45.9 (3.1)	< 0.001
South East-Asia, % (SE)	28,011	37.2 (1.1)	42.6 (1.2)	44.7 (4.2)	53.3 (3.0)	< 0.001
Western Pacific, % (SE)	39,697	51.6 (1.2)	54.5 (1.2)	54.7 (3.4)	53.5 (3.5)	0.113

*SE, standard error*.

### 3.3 Association between parental smoking and adolescent loneliness

After adjusting for age, sex, socioeconomic status, physical activity time, number of close friends, parental understanding, survey year, and country, parental smoking was positively associated with adolescent loneliness (neither as reference; father only: OR = 1.18, 95% CI = 1.10–1.26; mother only: OR = 1.39, 95% CI = 1.15–1.67; both: OR = 1.65, 95% CI = 1.41–1.93). In subgroup analyses by sex, age, and WHO region, the results were similar to the primary analysis (Table 3) except that the association was not significant in the African and Western Pacific regions. In the sensitivity analysis, similar results were observed overall and in the subgroups (by sex, age, and WHO region) according to the alternate definition of loneliness (Supplementary Table S3). Meanwhile, the association was not significant in adolescents with high frequency of tobacco use or alcohol consumption (Supplementary Tables S4, S5). The mediation analysis suggested that adolescent tobacco use and alcohol consumption partially mediated the association between parental smoking and adolescent loneliness, with the mediation proportion of 11.6% for adolescent tobacco use and 10.3% for adolescent alcohol consumption.

**Table 3:** Association between parental smoking and adolescent loneliness stratified by sex, age group, and WHO region.

	***N***	**Parental smoking**			
		**Neither**	**Father only**	**Mother only**	**Both**
**Model 1**					
**Total**	183,559	1.00	1.29 (1.20–1.39)	1.50 (1.26–1.79)	1.77 (1.53–2.04)
**Sex**					
Boys	86,334	1.00	1.23 (1.10–1.36)	1.47 (1.15–1.88)	2.03 (1.64–2.53)
Girls	97,225	1.00	1.36 (1.23–1.49)	1.52 (1.21–1.93)	1.49 (1.25–1.78)
**Age group, years**					
12–13	63,679	1.00	1.30 (1.16–1.45)	1.47 (1.14–1.90)	1.76 (1.39–2.22)
14–15	119,880	1.00	1.28 (1.18–1.40)	1.52 (1.21–1.89)	1.77 (1.48–2.13)
**WHO region**					
Africa	12,517	1.00	1.06 (0.84–1.35)	1.60 (0.92–2.76)	1.40 (0.94–2.10)
America	61,912	1.00	1.62 (1.41–1.86)	2.14 (1.68–2.72)	1.84 (1.40–2.43)
Eastern Mediterranean	44,342	1.00	1.19 (1.04–1.37)	1.43 (1.07–1.92)	1.85 (1.44–2.38)
South East-Asia	25,278	1.00	1.28 (1.14–1.43)	1.34 (0.94–1.92)	1.94 (1.51–2.50)
Western Pacific	39,510	1.00	1.11 (1.00–1.22)	1.12 (0.87–1.45)	1.02 (0.78–1.34)
**Model 2**					
**Total**	170,607	1.00	1.18 (1.10–1.26)	1.39 (1.15–1.67)	1.65 (1.41–1.93)
**Sex**					
Boys	79,852	1.00	1.14 (1.01–1.28)	1.37 (1.03–1.83)	1.85 (1.46–2.34)
Girls	90,755	1.00	1.20 (1.10–1.30)	1.37 (1.07–1.74)	1.40 (1.15–1.71)
**Age group, years**					
12–13	59,468	1.00	1.20 (1.07–1.34)	1.55 (1.19–2.02)	1.74 (1.34–2.25)
14–15	111,139	1.00	1.17 (1.06–1.28)	1.32 (1.03–1.68)	1.60 (1.32–1.94)
**WHO region**					
Africa	10,059	1.00	0.93 (0.72–1.20)	0.98 (0.52–1.84)	0.96 (0.61–1.51)
America	58,537	1.00	1.50 (1.31–1.72)	2.01 (1.57–2.58)	1.64 (1.25–2.14)
Eastern Mediterranean	42,130	1.00	1.22 (1.08–1.38)	1.62 (1.23–2.14)	1.99 (1.52–2.59)
South East-Asia	23,087	1.00	1.18 (1.05–1.33)	1.36 (0.93–2.05)	1.82 (1.39–2.38)
Western Pacific	36,794	1.00	1.10 (0.98–1.23)	0.89 (0.67–1.19)	1.02 (0.76–1.37)

*Data are presented as odds ratios (95% confidence intervals)*.

*Model 1: adjusted for age, sex, socioeconomic status*.

*Model 2: Model 1 covariates plus additional adjustment for physical activity, having a close friend, survey year, country, and parental understanding*.

*Hunger due to lack of food was not adjusted in Bhutan as this variable was not collected. Information on physical activity was not collected in Maldives and Malawi. In Cambodia parental understanding was not adjusted as information on this variable was not collected*.

## 4 Discussion

Of these 187,552 adolescents included in this study, 37% had at least one parental smoker, and nearly 40% felt lonely sometimes or more frequently in the past 12 months. After adjusting for all covariates, parental smoking was positively associated with adolescent loneliness. The association was stronger in adolescents whose parents were both smokers compared to those who had a single parent who was a smoker. In addition, the association was statistically significant in three WHO regions including regions of America, Eastern Mediterranean, and South East-Asia, rather than in regions of Africa and Western Pacific.

Our study offers important implications for mental health prevention via reducing passive smoking in LMIC adolescents. Previous studies have mainly examined the association between passive smoking and adolescent physical health. For example, it was reported that secondhand smoke exposure is associated with adolescent obesity among adolescents in LMICs (29). In addition, secondhand smoke exposure is associated with respiratory symptoms in adolescents from Hong Kong (30). Besides, our study showed that parental smoking can have a negative impact on the mental health of adolescents. Furthermore, our findings broaden the understanding of the health impacts of passive smoke exposure and reinforce the need to limit secondhand smoke exposure among adolescents.

Relevant studies on the association between parental smoking and adolescent loneliness are rare. To our knowledge, only two studies have focused on the association between passive smoking and adolescent loneliness (23, 31). One study including 191,613 non-smoking adolescents showed that exposure to secondhand smoke was positively associated with the likelihood of adolescent loneliness, with the odds of loneliness increasing with the number of days in the past 7 days exposed to secondhand smoke (0 days as reference; 1–2 days: OR = 1.11, 95% CI = 1.02–1.21; 3-−4 days: OR = 1.41, 95% CI = 1.27–1.57; 5-6 days: OR = 1.67, 95% CI = 1.44–1.93; 7 days: OR = 1.77, 95% CI = 1.60–1.96) (23). Another study including 9,143 adolescents from Caribbean countries showed that passive smoking was associated with adolescent loneliness (boys: OR = 1.28, 95% CI = 1.11–1.48; girls: OR = 1.21, 95% CI = 1.06–1.37) (31). These studies partly support our findings.

Previous studies have shown that parental smoking increases the likelihood of adolescent smoking (32, 33). In this study, the association between parental smoking and adolescent loneliness was not significant in adolescents who have high frequency of tobacco use or alcohol consumption. Our finding suggested that the association of parental smoking with adolescent loneliness was partly mediated by adolescent smoking or alcohol consumption. Previous studies have shown that the use of tobacco products and secondhand smoke exposure were associated with mental health issues such as depression and anxiety in adults (34, 35). Thus, parental smoking may influence adolescent mental health by affecting parental mental health. According to a Finnish cohort study, individuals exposed to parental smoking throughout childhood or adolescence had lower cognitive skills in midlife (RR = 1.38, 95% CI: 1.01–1.75) (36). This finding indicates that parental smoking during an individual's adolescence or childhood can have lifelong psychological consequences.

The potential explanation of the association between tobacco smoking and a sense of loneliness is that nicotine exposure can create more negative sentiments and a commensurate rise in adolescent loneliness (37, 38). It has been found that family smoking and exposure to secondhand smoke at home were risk factors for childhood unhappiness (39). In addition, exposure to secondhand smoke was associated with depressive symptoms, and suicidal ideation in Korean adolescents (40). These findings are consistent with our study. An additional explanation is that parental smoking behavior directly affects adolescent mental health. Nicotine can regulate the transmission of various neurochemicals in various mesocorticolimbic structures, which can affect mental health among adolescents (41). Additionally, both tobacco use and the use of E-cigarettes have been found to have adverse effects on the mental health of adolescents (42, 43). Although secondhand smoke exposure is decreasing among adolescents worldwide, adolescents with low socioeconomic status are still at increased odds of secondhand smoke exposure in public places and at home (2). Adolescents with smoking parents may have a lower socioeconomic status, making them at higher odds of loneliness. Additionally, adolescents whose parents smoke may face increased familial pressure, which can further contribute to feelings of loneliness among them. Our study findings suggest that maternal smoking has a more substantial effect on adolescent loneliness than paternal smoking. The stronger association between mothers and adolescents may play a crucial role in this phenomenon, in addition to the possibility that maternal smoking, as a central feature of the family environment, might contribute to a more hostile home environment (44).

Our study found that the association between single parental smoking and adolescent loneliness was stronger among girls than boys. This could be partly explained by the higher prevalence of tobacco use among boys, potentially normalizing smoking behavior within boys' social network, thereby mitigating feelings of loneliness (45). However, in our study, the association between both parental smoking and a sense of loneliness is stronger in boys than in girls. A plausible explanation is that smoking by both parents may create a high-stress family environment, which can have a greater impact on adolescent boys. Boys, who are more prone to risk-taking and externalizing behaviors, may be more sensitive to family stress and disadvantage than girls (46). Additionally, a global study of secondhand smoke exposure among adolescents, adolescents in regions of South-East Asia, Eastern Mediterranean, and Americas had a higher prevalence of secondhand smoke exposure at home on more than 5 days in the past 7 days than regions of Africa and the Western Pacific (2). This might explain the stronger association between parental smoking and loneliness among adolescents in these WHO regions.

This study has two strengths. First, to our knowledge, this is the first study to examine the association between parental smoking and adolescent loneliness based on a large sample size. Second, the same questionnaire and methodology were used in all GSHS participating countries, allowing the pooled analysis of data from all included countries. However, there are also some notable limitations. First, only students in low- and middle-income countries were surveyed and included, and the results may not be generalizable to adolescents in high-income countries. Second, the GSHS was conducted among students who were in school, and data may not be representative of adolescents in that country who were not in school for various reasons during the survey. Third, because GSHS is a cross-sectional survey, a causal relationship between adolescent parental smoking and adolescent loneliness should be made with caution. Fourth, other substance use that might confound the association were not adjusted in our study. Fifth, both exposure and outcome variables were collected via a self-report questionnaire, which may be subject to recall bias and desirability bias, potentially leading to inaccuracy in reporting. Sixth, insufficient effort responding (IER) is another potential limitation. The reliance on self-report data can increase the risk of IER, and respondents may provide careless or inattentive answers, leading to both random and systematic measurement errors. Given the standard procedures and quality control measurements implemented in the GSHS survey, we believe that the extent of IER in our study is likely to be mild. However, future research should adopt strategies to detect IER and reduce its potential impact on the results. Seventh, the evaluation of parental smoking in this study seemed to overlook ex-smokers, dosage, and environmental factors, thereby reducing the precision and granularity of the study. Eighth, while our study is the first to examine the association between parental smoking and adolescent loneliness in such a large sample, it is important to acknowledge the limitation of using a single-item measure to define constructs like parental smoking, as it may not fully capture the complexity of the variable.

## 5 Conclusion

Our study indicates that parental smoking might be an independent risk factor for adolescent loneliness, which is independent of many potential variables. Our finding expands the well-known physical damage of parental smoking in adolescents to the psychological damage. Thus, strict policies and strategies should be established to encourage smoking cessation for parents who are current smokers.

## Data Availability

The Global School-based Student Health Survey is a public dataset with de-identified information. The datasets presented in this study can be found in online repositories. The names of the repository/repositories and accession number(s) can be found below: Data are available from the websites of the United States Centers for Disease Control and Prevention (https://archive.cdc.gov/#/details?url=https://www.cdc.gov/gshs/index.htm).
