# Supplementary material for: Parental smoking is associated with adolescent loneliness: evidence from 71 low- and middle-income countries
Source: Front Public Health. 2025 Feb 26;13:1497136. doi: 10.3389/fpubh.2025.1497136 (PMC11896867; doi:10.3389/fpubh.2025.1497136)
Supplement: Supplementary file 1 [file Data_Sheet_1.pdf]

Table S1 Distributions of analyses' covariates by sex, age, and WHO region.

	Low socioeconomic status	Sufficient physical activity	Having close friends	Parental understanding	Frequency of tobacco use in the past 30 days			Frequency of drinking alcohol in the past 30 days		
					Never	0-9 days	≥10 days	Never	0-9 days	≥10 days
Total	10,366 (6.4)	28,706 (15.6)	164,723 (94.3)	67,852 (37.2)	145,355 （90.0）	15,438 （7.8）	5,099 （2.1）	99,458 (90.0)	26,358 (9.2)	3,345 (0.8)
Sex										
Boys	5,110 (6.8)	16,617 (18.5)	77,057 (94.4)	31,342 (36.8)	64,942 (85.9)	8,947 (11.2)	3,404 (3.3)	45,307 (89.0)	12,227 (10.0)	1,676 (1.0)
Girls	5,256 (6.0)	12,089 (12.6)	87,666 (94.3)	36,510 (40.1)	80,413 (94.7)	6,491 (4.4)	1,696 (0.9)	54,151 (90.9)	14,131 (8.3)	1,669 (0.7)
Age groups, years										
12-13	3,620 (6.0)	9,980 (14.5)	57,442 (94.6)	24,680 (38.9)	52,525 (92.4)	4,224 (6.3)	960 (1.3)	37,545 (93.1)	5,982 (6.3)	660 (0.6)
14-15	6,746 (6.7)	18,726 (16.3)	107,281 (94.1)	43,172 (38.1)	92,830 (88.5)	11,214 (8.8)	4,139 (2.6)	60,913 (87.9)	20,376 (11.1)	2,685 (1.0)
WHO Region										
Africa	1,200 (10.0)	1,843 (18.3)	9,279 (90.8)	3,899 (39.2)	8,693 (93.0)	825 (5.3)	289 (1.7)	6,914 (92.0)	1,503 (6.8)	183 (1.2)
America	1,971 (3.0)	10,420 (16.2)	56,224 (94.2)	25,183 (41.8)	50.174 (86.4)	5,826 (11.0)	1,855 (2.6)	33,899 (66.2)	19,631 (29.9)	2,785 (3.9)
Eastern Mediterranean	3,358 (6.9)	6,556 (12.8)	39,999 (92.9)	17,420 (38.0)	35,324 (90.4)	4,365 (7.6)	1,545 (2.0)	4,945 (92.3)	436 (7.0)	56 (0.7)
Southeast Asia	1,330	3,996	22,327	9,748	18,840	1,426	449	21,437	1,083	93

	(6.0)	(19.0)	(95.3)	(40.9)	(90.6)	(7.1)	(2.2)	(95.9)	(3.8)	(0.3)
Western Pacific	2,507<br>(6.5)	5,891<br>(9.2)	36,894<br>(95.9)	11,602<br>(29.4)	32,324<br>(88.4)	2,996<br>(9.7)	961<br>(1.9)	32,263<br>(85.4)	3,705<br>(14.2)	228<br>(0.4)

Data are presented as number (percentage).

Table S2 Prevalence of adolescent loneliness across different categories of parental smoking stratified by sex, age, and WHO region

	Parental smoking				*P*-value
	Neither	Father only	Mother only	Both	
**Total, % (SE)**	10.1(0.3)	12.2(0.6)	15.8(1.3)	18.4(2.1)	<0.001
**Sex**					
Boys, % (SE)	8.9(0.4)	10.2(0.9)	14.4(1.8)	19.7(3.2)	<0.001
Girls, % (SE)	11.4(0.4)	14.4(0.8)	17.6(1.8)	16.9(1.7)	<0.001
Age groups, years					
12-13, % (SE)	8.8(0.5)	10.1(0.8)	14.6(1.9)	18.8(3.0)	<0.001
14-15, % (SE)	10.9(0.3)	13.5(0.8)	16.5(1.7)	18.2(2.8)	<0.001
**WHO region**					
Africa, % (SE)	8.7(0.6)	10.6(1.9)	18.4(5.2)	17.9(4.3)	0.003
America, % (SE)	9.8(0.4)	15.5(1.2)	23.0(2.7)	20.4(2.5)	<0.001

Eastern Mediterranean, % (SE)	13.6(0.7)	15.8(1.4)	19.9(2.6)	18.8(2.9)	0.014
South East-Asia, % (SE)	7.3(0.5)	8.4(0.6)	8.3(1.5)	17.7(4.5)	<0.001
Western Pacific, % (SE)	12.9(0.8)	13.9(0.8)	13.6(3.1)	19.1(2.7)	0.107

SE=standard error.

Adolescent loneliness is defined as adolescents feeling lonely most of the time or always in the past 12 months.

Table S3 Association between parental smoking and adolescent loneliness stratified by sex, age group, and WHO region

	Parental smoking			
	Neither	Father only	Mother only	Both
**Model 1**				
**Total**	1.00	1.26 (1.13-1.39)	1.61 (1.34-1.94)	2.09 (1.62-2.70)
**Sex**				
Boys	1.00	1.19 (0.99-1.42)	1.70 (1.29-2.25)	2.69 (1.86-3.89)
Girls	1.00	1.32 (1.17-1.49)	1.52 (1.18-1.96)	1.54 (1.21-1.95)
**Age group, years**				
12-13	1.00	1.19 (1.03-1.38)	1.66 (1.21-2.27)	2.34 (1.69-3.22)
14-15	1.00	1.29 (1.14-1.47)	1.59 (1.26-2.00)	1.96 (1.36-2.83)
**WHO region**				
Africa	1.00	1.35 (0.97-1.88)	1.71 (0.93-3.13)	2.29 (1.34-3.91)
America	1.00	1.64 (1.35-1.99)	2.89 (2.13-3.91)	2.44 (1.67-3.56)

Eastern Mediterranean	1.00	1.21 (1.01-1.46)	1.48 (1.05-2.08)	1.48 (1.05-2.09)
South East-Asia	1.00	1.19 (1.01-1.41)	1.25 (0.85-1.84)	2.88 (1.63-5.10)
Western Pacific	1.00	1.13 (1.00-1.28)	1.35 (0.86-2.13)	1.69 (1.16-2.46)
**Model 2**				
**Total**	1.00	1.26 (1.14-1.40)	1.55 (1.28-1.90)	1.93 (1.43-2.60)
**Sex**				
Boys	1.00	1.20 (1.00-1.44)	1.57 (1.14-2.17)	2.33 (1.54-3.52)
Girls	1.00	1.29 (1.14-1.45)	1.48 (1.15-1.91)	1.50 (1.14-1.97)
**Age group, years**				
12-13	1.00	1.20 (1.03-1.39)	1.72 (1.23-2.41)	2.33 (1.59-3.41)
14-15	1.00	1.30 (1.14-1.48)	1.49 (1.17-1.91)	1.73 (1.13-2.64)
**WHO region**				
Africa	1.00	1.11 (0.73-1.67)	1.63 (0.83-3.22)	1.71 (0.97-3.03)
America	1.00	1.51 (1.22-1.87)	2.46 (1.78-3.42)	2.07 (1.49-2.89)

Eastern Mediterranean	1.00	1.25 (1.05-1.50)	1.73 (1.21-2.48)	1.54 (1.05-2.26)
South East-Asia	1.00	1.31 (1.08-1.58)	1.15 (0.75-1.77)	2.60 (1.33-4.93)
Western Pacific	1.00	1.07 (0.95-1.22)	0.97 (0.57-1.65)	1.49 (0.99-2.22)

Data are presented as odds ratios (95% confidence intervals)

Adolescent loneliness is defined as adolescent feeling lonely most of the time or always in the past 12 months

Model 1: adjusted for age, sex, and socioeconomic status.

Model 2: Model 1 covariates plus additional adjustment for physical activity, having a close friend, survey year, country, and parental understanding.

Note: Hunger due to lack of food was not adjusted in Bhutan as this variable was not collected. Data on physical activity were not collected in Maldives and Malawi. Cambodia was not adjusted for parental understanding as these variables were not collected.

Table S4 Associations between parental smoking and adolescent loneliness, stratified by frequency of tobacco use and alcohol consumption

	N	Parental smoking				Proportion of mediation, %	*P* value
		Neither	Father only	Mother only	Both		
Frequency of tobacco use in the past 30 days						11.6	<0.001
Never	145,355	1.00	1.21 (1.13-1.30)	1.17 (0.91-1.50)	1.49 (1.21-1.82)		
1-9 days	15,438	1.00	0.97(0.76-1.24)	1.31(0.92-1.87)	1.67(0.98-2.85)		
≥10 days	5,099	1.00	0.84(0.46-1.51)	1.34 (0.60-2.99)	1.57(0.74-3.31)		
Frequency of drink alcohol in the past 30 days						10.3	<0.001
Never	99,458	1.00	1.15 (1.05-1.26)	1.35 (1.00-1.82)	1.54 (1.26-1.88)		
1-9 days	26,358	1.00	1.12(0.93-1.35)	0.91(0.60-1.35)	1.21(0.84-1.75)		
≥10 days	3,345	1.00	1.49(0.57-3.90)	1.14(0.43-3.02)	2.82(0.79-10.01)		

Data are presented as odds ratios (95% confidence intervals)

Model was adjusted for age, sex, socioeconomic status, physical activity, having a close friend, parental understanding, survey year, and country.

Note: Afghanistan, Bahrain, Egypt, Iraq, Kuwait, Morocco, Pakistan, Palestine, Qatar, United Arab Emirates, and Yemen did not collect information on alcohol consumption, and this variable was not considered for these countries. In addition, Myanmar did not collect information on tobacco use, and this variable was not considered for this country.

Table S5 Sensitivity analysis of associations between parental smoking and adolescent loneliness[*], stratified by frequency of tobacco use and alcohol consumption

	N	Parental smoking				Proportion of mediation, %	*P* value
		Neither	Father only	Mother only	Both		
Frequency of tobacco use in the past 30 days						10.7	<0.001
Never	145,355	1.00	1.28 (1.14-1.45)	1.26 (0.95-1.66)	1.46 (1.06-2.01)		
1-9 days	15,438	1.00	0.93(0.67-1.28)	1.54(1.02-2.31)	2.09(1.01-4.33)		
≥ 10 days	5,099	1.00	1.03(0.57-1.87)	0.86(0.38-1.93)	1.46(0.79-2.69)		
Frequency of drink alcohol in the past 30 days						20.4	0.004
Never	99,458	1.00	1.23 (1.08-1.39)	1.39(1.06-1.83)	2.06 (1.31-3.24)		
1-9 days	26,358	1.00	1.11(0.89-1.39)	0.91(0.56-1.47)	1.78(1.11-2.85)		
≥ 10 days	3,345	1.00	2.00(0.86-4.77)	2.91(0.95-8.91)	2.31(0.98-5.43)		

Data are presented as odds ratios (95% confidence intervals)

[*] Adolescent loneliness is defined as adolescent feeling lonely most of the time or always in the past 12 months

Model was adjusted for age, sex, socioeconomic status, physical activity, having a close friend, parental understanding, survey year, and country.

Note: Afghanistan, Bahrain, Egypt, Iraq, Kuwait, Morocco, Pakistan, Palestine, Qatar, United Arab Emirates, and Yemen did not collect information on alcohol consumption, and this variable was not considered for these countries. In addition, Myanmar did not collect information on tobacco use, and this variable was not considered for this country.
